# Exploring Cannabinoid Effects Using Zebrafish (*Danio rerio*) as an In Vivo Model: A Review of the Literature

**DOI:** 10.3390/ijms26189165

**Published:** 2025-09-19

**Authors:** Xingbo Wang, Han Xie, Xiaoling Shi, Kusheng Wu, Wenlong Huang

**Affiliations:** 1 Department of Forensic Medicine, Shantou University Medical College, Shantou 515041, China; 24xbwang@stu.edu.cn; 2 Department of Preventive Medicine, Shantou University Medical College, Shantou 515041, China; 23hxie@stu.edu.cn (H.X.); 20xlshi@stu.edu.cn (X.S.); kswu@stu.edu.cn (K.W.)

**Keywords:** cannabinoids, therapeutic potential, toxicological profiles, zebrafish

## Abstract

Cannabis is increasingly utilized for both recreational and medical purposes, and the discovery of the endocannabinoid system (ECS) has renewed interest in its therapeutic potential. Nonetheless, the safety of cannabis and cannabinoid-containing products requires re-evaluation. In this study, zebrafish were employed as a translational in vivo model to comprehensively evaluate the toxicological profiles and the therapeutic potential of phytocannabinoids and synthetic cannabinoids. Current evidence, particularly from studies on key phytocannabinoids such as Δ^9^-THC, CBD, and CBN, along with newly developed synthetic cannabinoids (such as JWH-018), demonstrates a spectrum of embryotoxic outcomes including developmental abnormalities, neurotoxicity, liver damage, reproductive impairments, and disturbances in metabolic regulation, especially during early life stages. By contrast, evidence for therapeutic benefits, such as alleviation of muscle spasms, pain and nausea, as well as neuroprotective and anti-inflammatory effects, is promising but comparatively less abundant and more heterogeneous in study design and outcome measures. Taken together, this imbalance indicates that toxicological risks are supported by more extensive and consistent data, whereas therapeutic efficacy, though encouraging, still requires more rigorous validation. This dual profile underscores the need for a robust, evidence-based framework for cannabinoid development and clinical application. Further investigations are essential to clarify mechanisms of toxicity and therapeutic action, optimize dosing regimens, define safe therapeutic windows, and evaluate long-term health outcomes.

## 1. Introduction

*Cannabis sativa* L. (*C. sativa*, family: *Cannabaceae*), commonly known as marijuana or Indian hemp, is a versatile plant with diverse applications in medicine, nutrition, and industry, despite its controversial legal status and potential health concerns. It contains over 560 chemicals, including approximately 125 cannabinoids, 200 terpenes, alkaloids and various flavonoids [1]. For nearly 5000 years, cannabis extracts have been used for pain management and other therapeutic purposes [2]. The medicinal efficacy of Cannabis was first documented in the ancient Chinese text Shen Nong Ben Cao Jing, highlighting its long-standing therapeutic significance. Today, the World Health Organization (WHO), reports that approximately 147 million people, or 2.5% of the global population, use cannabis annually [3]. This can be explained by the increasing acceptance and legalization of cannabis for medical and recreational purposes across multiple countries [4].

The growing prevalence of cannabis use, alongside the emergence of synthetic cannabinoids, has stimulated research into their potential toxicological effects and health risks. Existing studies have reported diverse outcomes, including developmental, neurotoxic, hepatotoxic, reproductive, and metabolic effects, but results are often heterogeneous and limited in scope. Furthermore, while some evidence points to therapeutic benefits, such as analgesic, anti-inflammatory, and neuroprotective effects, comprehensive evaluation of efficacy and safety remains lacking.

In this context, zebrafish (*Danio rerio*) have emerged as a powerful translational model to systematically assess cannabinoid toxicity and therapeutic potential. Their genetic similarity to humans, rapid development, and amenability to high-throughput screening facilitate in vivo evaluation of phenotypic and functional endpoints. This review aims to synthesize current evidence on the toxicological profiles and therapeutic effects of phytocannabinoids and synthetic analogs, highlighting knowledge gaps and guiding future research priorities.

## 2. Types of Cannabinoids

**Figure 1:**
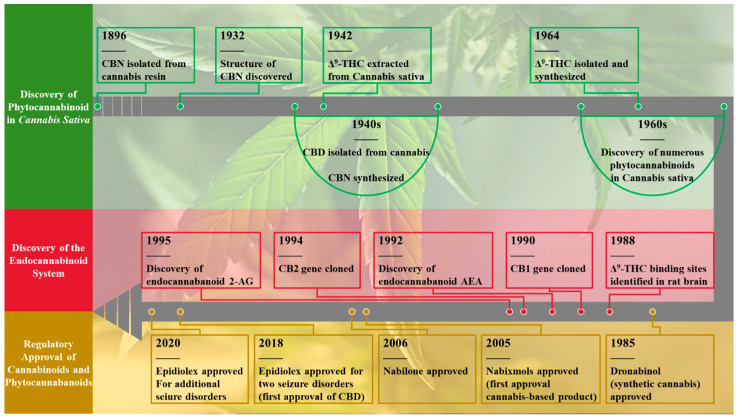
Timeline of cannabinoid discovery, isolation, and regulatory approval. Key elements: Δ^9^-THC (Δ-9-tetrahydrocannabinol), CBD (cannabidiol), CBN (cannabinol), AEA (anandamide; N-arachidonoylethanolamine), 2-AG (2-arachidonoylglycerol), CB1 (cannabinoid receptor type 1), CB2 (cannabinoid receptor type 2). Adapted with permission from Tycko Medical Art (Copyright © 2022).

The terms “cannabis,” “cannabinoids,” “cannabis-based medicinal products, cannabis-based therapeutic drugs”, “cannabis-derived extracts” and “medical cannabis” are often used interchangeably but they represent distinct concepts. Cannabis refers to the whole plant (*Cannabis sativa* L.) or its parts, whereas cannabinoids are chemically defined compounds, either plant-derived (phytocannabinoids), synthetic, or semi-synthetic, that typically bind to and activate cannabinoid receptors. Cannabis-based medicines encompass formulations containing cannabis extracts or purified cannabinoids. Based on their sources (Table 1), cannabinoids can be classified as endocannabinoids (ECs, or called eCB) produced by animal cells from polyunsaturated fatty acids; Phytocannabinoids (PCs), derived primarily from the cannabis plant; and synthetic cannabinoids (SCs), designed to mimic the effects of natural cannabinoids. These distinctions are critical for understanding their biological and therapeutic roles. Figure 1 presents a timeline chronicling the discovery, isolation, and regulatory authorization of cannabis-based therapeutic agents, detailed in the following section.

### 2.1. Phytocannabinoids (PCs)

PCs, a diverse group of naturally occurring compounds derived from Cannabis sativa, have been extensively studied for their therapeutic potential. They primarily consist of 21-carbon terpenophenolics (or 22-carbon in their acidic forms), encompassing over 120 identified variants. The most prominent include delta-9-tetrahydrocannabinol (Δ^9^-THC, commonly known as “THC”), cannabidiol (CBD), cannabigerol (CBG), and cannabichromene (CBC), collectively referred to as the “Big Four” [5]. These compounds are extensively studied for their biological activity and are more readily synthesized compared to other PCs [6]. PCs are classified into psychotropic and non-psychotropic, among these. THC, the primary psychoactive compound, was first described in the 1940s [7] and fully characterized in 1964 [8]. Non-psychoactive cannabinoids include cannabinol (CBN), cannabitriol (CBT), and cannabinodiol (CBND). CBN isolated in 1896 by Wood and colleagues, is a non-enzymatic oxidative breakdown product of THC due to aging or light exposure [9]. Among these, CBD stands out as the most pharmacologically promising compound due to its significant therapeutic potential and lack of psychoactive effects, though its efficacy may vary by cannabis strain. CBD has gained widespread global popularity and is commercially available in various forms, including dietary supplements, creams, lotions, and, most commonly, oils. Overall, these compounds have shown promise in various pharmacological applications, driving interest in their medicinal use [10]. For instance, nabiximols (oromucosal spray, marketed as Sativex), a 1:1 mixture of THC: CBD, was approved in 2005 by Health Canada for managing neuropathic pain and spasticity in multiple sclerosis [11]. Sativex is available in many other countries, for example, in Spain, France, and the UK, but not FDA-approved in the US. Epidiolex, a plant-derived CBD drug, is approved in 2018 by the FDA for the treatment of seizures associated with Dravet and Lennox–Gastaut syndromes, in patients two years and older (Figure 1 and Table 2) [12].

### 2.2. Endogenous Cannabinoids or Endocannabinoids (eCB, or ECs)

The first described eCB is arachidonylethanolamine (AEA), also known as anandamide, isolated from the porcine brain in 1992 [13]. The term “anandamide” derived from the Sanskrit word “ananda”, meaning “internal bliss” or “joy”. The second EC identified is 2-arachidonoyl glycerol (2-AG). AEA and 2-AG, both derivatives of arachidonic acid, are the principal endogenous ligands of the classical endocannabinoid signaling system (ECS). The ECS composes eCBs, cannabinoid receptors (CB1, CB2) and related receptors (e.g., TRPV, PPARs), enzymes such as FAAH and MAGL responsible for ECs synthesis and degradation, and genes encoding these receptors and enzymes. The ECS was discovered in 1988 by Allyn Howlett and W.A. Devane [14,15], and plays critical roles in regulating multiple physiological systems in humans, including pain regulation, neurogenesis, immune modulation and energy homeostasis.

### 2.3. Synthetic Cannabinoids (SCs)

SCs, first developed in the 1970s to study the ECS for pharmaceutical purposes, are commonly referred to as “synthetic marijuana” or “cannabimimetics” [16]. They bind to cannabinoid receptors, and mimic the effects of THC. Some of the SCs are marketed as medicines in several countries. For example, Nabilone (synthetic THC, marketed in the USA as Cesamet) and Dronabinol (synthetic THC, marketed in the USA as Marinol and Syndros) are approved for pharmaceutical use in the treatment of nausea and pain associated with cancer chemotherapy in several countries (Figure 1 and Table 2) [17].

These substances are typically sold as mixtures and marketed under various brand names, such as “Spice”, “K2”, “Black Mamba”, “Blue Lotus”, “Kronic”, “Moon Rocks”, “Dream”, “Zombie”, “Sence” and “Mr. Nice Guy”, often labeled as “herbal incenses”, “herbal smoking mixtures” or “for aromatherapy only” to evade regulatory oversight [18]. SCs are categorized into seven groups based on their chemical structure: naphthoylindoles, naphthylmethylindoles, naphthoylpyrroles, naphthylmethylindenes, phenylacetylindoles, cyclohexylphenols, and dibenzopyrans. Over 450 SCs have been developed, and their non-medical use has surged among adolescents and young adults due to curiosity, affordability, and perceived psychoactive effects. However, these SCs can be unpredictable and dangerous for users. Their widespread misuse and ever-changing molecular structure to bypass regulations have led to significant public health and social challenges, including adverse health outcomes and substance abuse concerns. Additionally, SCs and their metabolites can contaminate aquatic ecosystems through wastewater, posing risks to human health via drinking water and the food chain.

### 2.4. Cannabinoid Signaling Pathways and Effects

**Figure 2:**
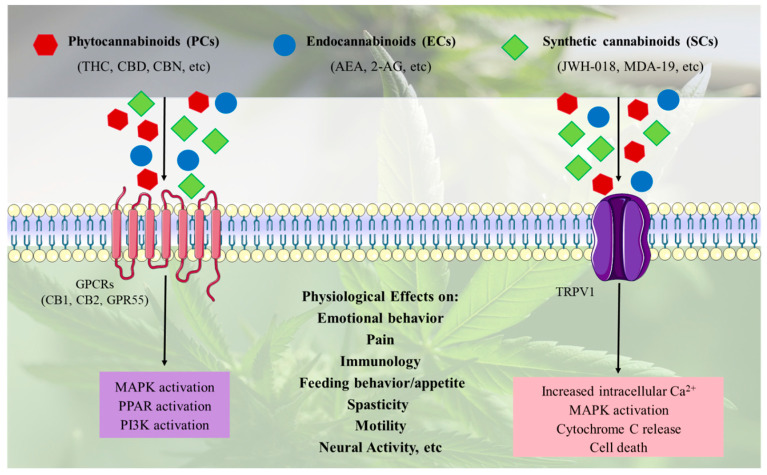
PCs, ECs and SCs exert their effects by interacting with G-protein coupled receptors (GPCRs) such as CB1, CB2, and G protein-coupled receptor 55 (GPR55), or ion channels like transient receptor potential vanilloid 1 (TRPV1). This interaction triggers various cellular responses that influence a wide range of physiological processes, including emotional behavior, depression, nervous functions, neurogenesis, anxiety, feeding behavior/appetite, reward, cognition, learning, memory.

PCs, EC, and SCs exert their effects by binding to cannabinoid receptors on cell membranes, primarily the G protein-coupled receptors CB1 and CB2, as well as other receptors such as G protein-coupled receptor 55 (GPR55), peroxisome-proliferator-activated receptor (PPAR), and transient receptor potential vanilloid 1 (TRPV1) ion channel. The signaling pathways activated by these receptors vary depending on the receptor type and the specific cannabinoid ligand. For instance, CB1 and CB2 activation typically inhibits adenylyl cyclase via Gi/o proteins, reducing cyclic AMP (cAMP) levels and modulating downstream pathways such as MAPK and ion channel activity. ECs, such as AEA and 2-AG, act as retrograde messengers to fine-tune synaptic activity, while SCs often exhibit higher binding affinity and prolonged activation, leading to more intense physiological effects. These effects include analgesia, appetite stimulation, mood alteration, and, in some cases, adverse outcomes like anxiety or psychosis, particularly with SCs due to their potency (Figure 2).

## 3. Zebrafish as a Translational Model for Cannabinoids-Induced Toxicological Profiling and Therapeutic Potential Evaluation

Zebrafish (*Danio rerio*; formerly *Brachydanio rerio*) was first established as a model organism for genetic research by Streisinger in the early 1980s [19]. It serves as a key model organism in toxicological research, well-suited for exploring cannabinoid toxicity and therapeutic potential within the One Health framework. Its rapid developmental cycle, high-throughput capabilities, embryonic and larval transparency, synchronous development, and cost-effectiveness meet the demands of modern toxicology, which embraces the 3Rs (Replacement, Reduction, Refinement) concept while reducing time and costs. The Fish Embryo Acute Toxicity (FET) test, detailed in OECD Test Guideline (TG) 236, evaluates the acute toxicity of chemicals using zebrafish embryos.

The highly conserved endocannabinoid system in zebrafish, closely resembling that of humans, strengthens its relevance as a model [20]. Additionally, an expanding range of larval disease models, including seizures, bipolar disorder, anxiety, and neurohyperactivity, enables robust testing of therapeutic interventions for PCs and SCs. Thus, zebrafish provide a powerful, efficient platform for advancing cannabinoid research.

### 3.1. Human Cannabinoid Receptors

**Figure 3:**
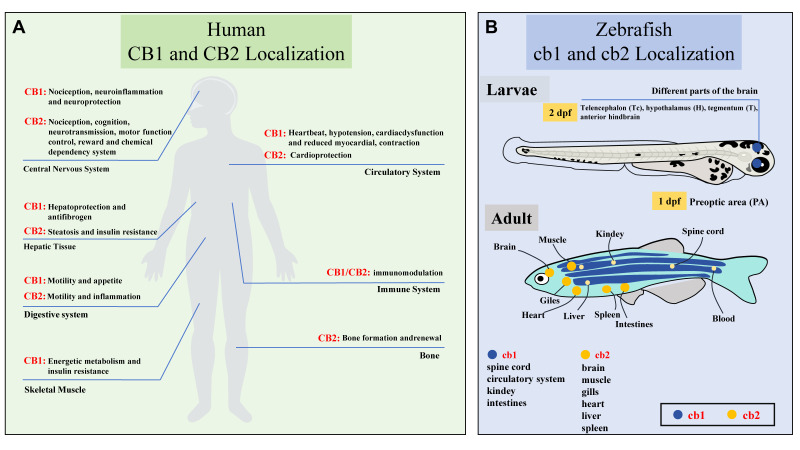
Endocannabinoid Receptor System. (**A**) In human, CB1 receptors exhibit prominent expression in the cerebral cortex, hippocampus, hypothalamus, cerebellum, spinal cord, and dorsal root ganglia, with additional presence in the enteric nervous system, adipocytes, endothelial cells, hepatocytes, muscle, and gastrointestinal tract. In contrast, CB2 receptors are primarily localized to immune tissues, including T and B cells, spleen, tonsils, and activated microglial cells. (**B**) In zebrafish, cb1 expression initiates in the pre-optic area at 1 dpf. During the late larval stage, cb1 expands to multiple brain regions including the telencephalon, hypothalamus, tegmentum, and anterior hindbrain. Adult tissues exhibit broad cb2 mRNA distribution, localized to the gills, heart, intestine, muscle, spleen, and central nervous system. Image adapted from [20].

CB1 and CB2 are the most well-characterized cannabinoid receptors. CB1 receptor (also known as Cnr1 or CB1R) is predominantly expressed in the brain, including regions such as the hippocampus, cerebellum, basal ganglia, and cerebral cortex. They are also present in various other tissues, such as the cervical ganglion, peripheral autonomic nerves, testes, spleen, peripheral leukocytes, sperm, uterus, vascular endothelial cells, smooth muscle cells, eye, and placenta. CB2 receptor (also known as Cnr2 or CB2R) are mainly expressed in the immune and hematopoietic systems, such as T and B cells, neutrophils, epithelial cells, and macrophages, modulating immune responses and inflammation (Figure 3A). CB1 was first identified in 1988 by Devane et al. [15], and CB2 was characterized in 1990 by Munro et al. [21]. Molecular cloning of CB2 receptors was accomplished by Munro et al. in 1993 [21]. Both receptors couple to Gi/o proteins, with CB1 linked to psychoactive effects and pain modulation, and CB2 associated with anti-inflammatory effects. Emerging evidence suggests CB2 presence in the CNS under certain conditions, broadening its therapeutic potential.

### 3.2. Zebrafish Cannabinoids Receptors

**Figure 4:**
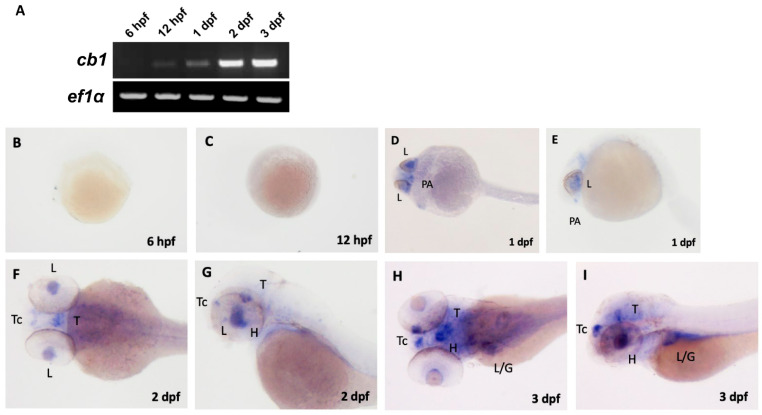
Reverse transcription PCR (RT-PCR) and in situ hybridization analysis of cb1 in embryo-larvae. (**A**) RT-PCR showing temporal expression profiles of *cb1* and reference gene *ef1α* at 6 h post fertilization (hpf), 12 hpf, 1 day post fertilization (dpf), 2 dpf, and 3 dpf. (**B**,**C**) In situ hybridization revealed no detectable cb1 expression at 6 hpf and 12 hpf. (**D**,**E**) At 1 dpf, expression localized to the pre optic area (PA) and lens (L). (**F**,**G**) By 2 dpf, expression expanded to the telencephalon (Tc), tegmentum (T), lens (L), and hypothalamus (H). (**H**,**I**) At 3 dpf, expression persisted in the telencephalon, tegmentum, and hypothalamus, with additional signals in the liver/gut region. This image is reproduced with permission from Refs. [23,24], 2022 S. Karger AG, Basel, Switzerland.

**Figure 5:**
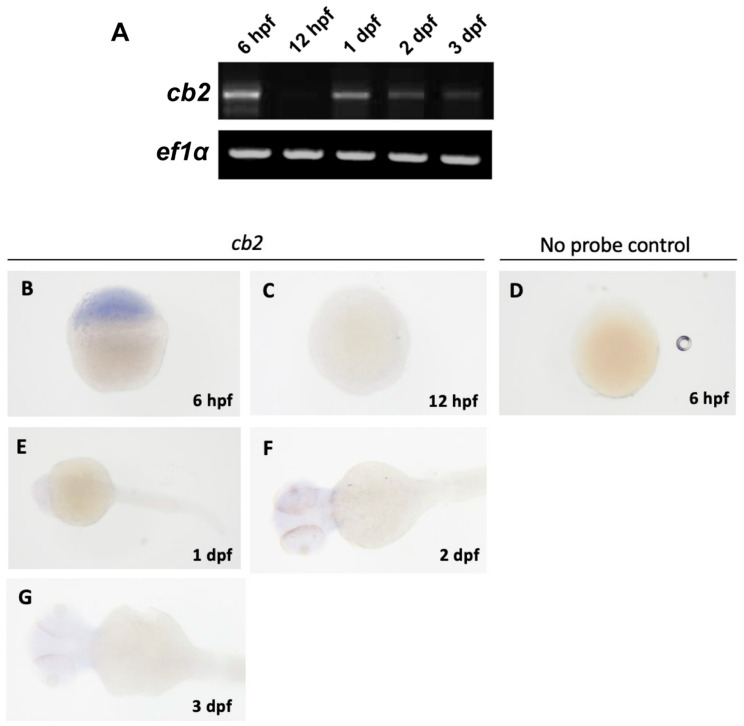
Expression analysis of cb2 by RT-PCR and in situ hybridization in zebrafish. (**A**) RT-PCR showing temporal expression of *cb2* and *ef1α* at 6, 12 hpf, 1, 2, and 3 dpf. (**B**) In situ hybridization detected cb2 expression at 6 hpf, (**C**) whereas no signal was observed at 12 hpf. (**D**) No-probe control at 6 hpf confirmed absence of non-specific staining. (**E**–**G**) Expression remained undetectable from 1 to 3 dpf in all examined tissues. This image is reproduced with permission from Refs. [23,24], 2022 S. Karger AG, Basel.

In zebrafish, cbs are present from very early developmental stages. Analysis of whole-larval homogenate reveals that cb1 mRNA is expressed from the 3-somite stage to the 25-somite stage [22]. Quantifying mRNA levels by whole-mount in situ hybridization, as shown in Figure 3B and Figure 4, cb1 is expressed in the preoptic area (PA) as early as 1 day post-fertilization (dpf). At later larval stages (e.g., 2–3 dpf), cb1 expression is detected in various brain regions, including the telencephalon (Tc), hypothalamus (H), tegmentum (T), and anterior hindbrain (AH) [23,24]. Oltrabella et al. reported a high level of cb2 mRNA expression as early as 4 hpf [25]. The expression patterns differ is that cb2 expression peaks within the first 24 h of development and then declines, whereas cb1 expression is low at 24 hpf but increases significantly by 48 hpf (Figure 5) [24,25]. In adult zebrafish, cb1 mRNA expression is more pronounced in the medial pallium of the dorsal telencephalon than in other brain regions [26].

Cannabinoid receptors are highly conserved across species. For example, the zebrafish cb1 protein shares 69% sequence identity and 73.6% amino acid identity with human CB1, while zebrafish cb2 shares only 39% amino acid identity with human CB2. These similarities, combined with the genetic and physiological advantages of zebrafish, make them an excellent model organism for studying the mechanisms of cannabis and its derivatives.

## 4. Searching Strategy

A literature review was performed in March to July 2025 using major electronic databases, including PubMed, Science Direct, Web of Science, ACS Publications, and Springer Link. A comprehensive list of relevant keywords and phrases, for example “zebrafish”, “zebra *danio*”, “*Danio rerio*”, “*Brachydanio rerio*” combined with “cannabinoid”, “delta-9-tetrahydrocannabinol (THC)”, “cannabidiol (CBD)”, “cannabigerol (CBG)”, “cannabichromene (CBC)”, “cannabinol (CBN)”, “synthetic cannabinoid (SCs)” was developed.

Titles and abstracts were screened to ensure relevance to the toxicity and therapeutic potential of PCs and SCs in zebrafish model, excluding studies focused on ECs. Reference lists of the included studies were also reviewed to identify additional relevant publications. Following the application of inclusion and exclusion criteria (e.g., peer-reviewed studies in English focusing on zebrafish toxicology of exogenous cannabinoids, metabolism of PCs or SCs using zebrafish model), a total of 44 publications were retained. It should be noted that all screening was conducted by a single reviewer, which may introduce a risk of selection bias.

For each eligible study, we extracted key experimental information, including exposure concentrations, initial exposure stage, exposure durations, and toxicological/therapeutic endpoints, such as morphological abnormalities, behavioral alterations, and relevant molecular changes. This approach enabled us to generate a comprehensive overview of how different cannabinoids have been examined in zebrafish across diverse developmental stages and experimental contexts.

**Figure 6:**
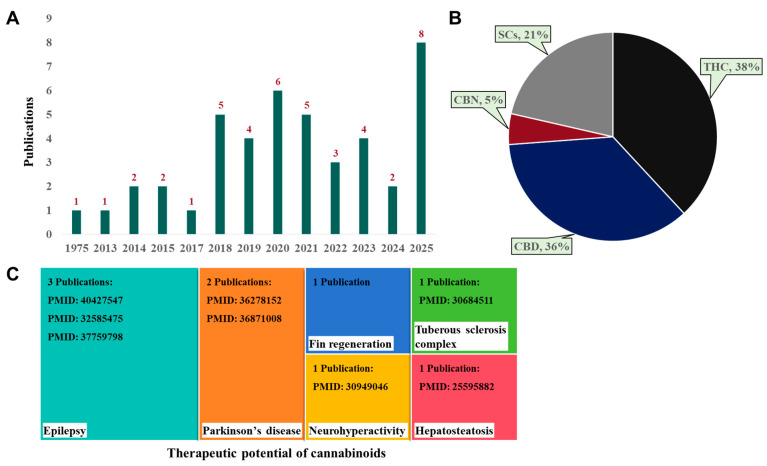
(**A**) Number of publications studying toxicity profiles and/or therapeutic potential of cannabinoids using zebrafish (*Danio rerio*) as a model organism (the last search for publications was carried out in July 2025). (**B**) Proportion of studies assessing toxicity profiles of THC, CBD, CBN, and SCs. (**C**) Number of publications exploring the therapeutic potential of cannabinoids in zebrafish disease models.

Figure 6 illustrates the number and proportion of publications studying the toxicity and therapeutic potential of cannabinoids. As shown in Figure 6A, the earliest studies using zebrafish embryos/larvae to investigate cannabinoids were conducted in 1975 [27]. Figure 6B depicts the proportion of studies assessing the toxicity profiles of PCs and SCs, with THC accounting for the largest share (38%), followed by CBD (36%), SCs (21%), and CBN (5%) in this review. Figure 6C presents the number of publications exploring the therapeutic potential of cannabinoids. These studies highlight the increasing focus on both the toxicological and therapeutic aspects of cannabinoids.

## 5. Effect of PCs Using Zebrafish as an In Vivo Model

In this section, we evaluate the toxicity profiles of THC, CBD, and CBN using the zebrafish model. Their effects in the zebrafish model are summarized in Table 3, Table 4 and Table 5, which compile data from multiple studies on various endpoints.

### 5.1. Toxicity of THC

THC, the best-characterized and the most psychoactive compound, is the most extensively studied of the “big four”. As presented in Table 4, this review includes 16 studies that investigate THC’s toxicity, focusing on developmental adversities, behavioral effects, neurotoxicity, and intergenerational toxicity.

#### 5.1.1. Developmental Toxicity of THC

The initial toxicity testing of THC using zebrafish larvae revealed that 1.0–10.0 ppm THC results in a reduction in spontaneous tail muscle twitch and induces distal trunk anomalies [27]. A bent body axis first appeared at concentrations of 0.3 mg/L and higher of THC, and pericardial and yolk sac edema can be seen at 0.6 mg/L or higher [29]. Furthermore, increased incidence of bent body axis and bent tail were found in embryos exposed chronically to THC. Decreased fecundity, THC can cause significant effects on longevity and health span of zebrafish in a biphasic manner [41].

#### 5.1.2. Behavioral Effects and Neurotoxicity of THC

This section examines the neurodevelopmental and locomotor impacts of THC exposure during critical developmental windows, specifically gastrulation (5.25–10.75 hpf) and larval stages (5 dpf), across varying doses and exposure durations.

Gastrulation-stage exposure: THC exposure during gastrulation (5.25–10.75 hpf, ~5-h exposure) impairs neurodevelopment, leading to persistent locomotor deficits. For example, THC exposure causes transient hyperactivity followed by sustained hypoactivity dose-dependently [39]. It also reduces spontaneous coiling in 1-dpf embryos and decreases both touch-evoked and basal swimming in 5-dpf larvae [47]. Structural analyses show reduced axonal branching in trunk musculature and diminished sensorimotor responses [46]. At 6 mg/L, THC causes Mauthner cell (M-cell) abnormalities, including reduced axonal diameter, and global neuronal hypoactivity, as detected by antibody-specific labeling (anti-3A10/RMO44) [42]. Furthermore, functional studies using CaMPARI zebrafish demonstrate that 4–6 mg/L THC (0.5–10 hpf) suppresses neuronal activation, with effects persisting until 4 dpf [44].

Acute THC exposure (0.3–2.4 mg/L; 1 or 12-h, starting at 108 hpf) induces a concentration-dependent biphasic locomotor response in zebrafish during the dark phase, characterized by hyperactivity at low concentrations and suppression at high concentrations. Additionally, a 0.5 h exposure to 10 mg/L THC at 5 dpf disrupts neuromuscular transmission, increasing the frequency of miniature endplate currents (mEPCs), and impairing locomotor responses [45]. Chronic THC exposure (96 h from 24 hpf) leads to habituation in basal and recovery phases across all concentrations, though 1.2 mg/L stimulates activity [29]. Furthermore, a 24 h exposure to 1.26 mg/L THC starting at 5 dpf (larval-stage exposure) reduces total distance traveled by 35.2% decrease at 6 dpf [43]. Low-dose THC exposure (0.024 mg/L, from ~6 hpf) also cause dark-phase hypoactivity at 96 hpf [41]. Moreover, exposure to 0.6 mg/L THC (starting at ~6 hpf) alters neurodevelopmental biomarkers: c-fos expression increases at 14 hpf but decreased at 48/72 hpf, while bdnf shows abnormal upregulation at 48 hpf and 96 hpf [41].

Taken together, THC exposure during critical windows (gastrulation or larval stages) induces time- and dose-dependent neural alterations. Specifically, gastrulation-stage exposure primarily causes structural defects, such as axon loss and M-cell damage, whereas acute or chronic larval-stage exposure results in functional dysfunction, including neuronal suppression and synaptic overactivity. These perturbations manifest as distinct locomotor impairments, including hypoactivity, hyperactivity, and disrupted sensorimotor integration, with effects heavily dependent on exposure timing, duration, and concentration.

#### 5.1.3. Multigenerational Effects of THC Exposure

THC has been demonstrated to cross the placental barrier, and its administration during pregnancy may disrupt ECS signaling in the developing fetus, which may contribute to long-term neurodevelopmental and behavioral outcomes in offspring [61,62]. In zebrafish, parental THC exposure (0.12 mg/L) results in transgenerational effects, significantly increasing locomotor activity in F1 larvae at 96 hpf [41]. However, another study found that parental THC exposure at concentrations of 0.001, 0.01, 0.1, and 0.5 mg/L reduces spontaneous coiling in F1 embryos at 1 dpf and touch-evoked swimming in F1 larvae at 2 dpf, while basal swimming at 5 dpf remains unaffected [47]. Additionally, early-life exposure to 2 µM THC (approximately 0.63 mg/L) induce lower survival rates and completely reproductive failure in the F1 generation [40]. These findings highlight that THC exposure causes diverse transgenerational effects in zebrafish, altering development, behavior, and reproduction across generations.

Collectively, these studies emphasize THC’s potential to disrupt embryonic development, alter locomotor activity, and induce neurological changes in zebrafish larvae, with some evidence suggesting that parental exposure may influence offspring phenotypes. Such findings underscore the importance of understanding the long-term and cross-generational impacts of this compound.

### 5.2. Toxicity of CBD

CBD, the most abundant non-euphoric PC derived from hemp, exhibits well-documented anti-inflammatory and antioxidant properties and is considered non-addictive [63]. While recent studies highlight its therapeutic potential, its effects during early development remain a key research focus. This review includes 15 studies, with 13 addressing developmental outcomes (e.g., developmental toxicity, behavioral and neurotoxic effects, hepatotoxicity, and reproductive toxicity), as summarized in Table 5.

#### 5.2.1. Behavioral Effects and Neurotoxicity of CBD

Gastrulation-stage exposure to 3 mg/L CBD reduces survival rates, body lengths, and heart rates and causes mild deformities. It also suppresses the frequency of mEPC activity at neuromuscular junctions (NMJs), alters branching patterns of secondary motor neurons, and changes synaptic nAChRs associated with skeletal musculature, and reduces response rates to sound stimuli [46]. These morphological anomalies are linked to inhibition of Shh signaling, as evidenced by reduced ptch2 expression and locomotor deficits. Co-treatment with the Smo agonist, purmorphamine mitigates these teratogenic effects, including decreased mortality, increased hatching rates, elevated ptch2 expression, and increased locomotor activity [56].

Using in vivo calcium imaging with the CaMPARI zebrafish, Kanyo et al. demonstrates that acute CBD exposure (0.5–10 hpf) persistent attenuates neural circuit activity, as evidenced by reduced neuronal activation at 4 dpf [44]. Furthermore, CBD-induced neurotoxicity was partially mediated through cb1 and cb2 receptors, with co-exposure to THC synergistically enhancing these neurotoxic effects [44].

#### 5.2.2. Hepatotoxicity of CBD

The hepatotoxicity of CBD has been extensively reported in clinical trials and research studies (see review [64,65]). In zebrafish, exposure to 0.01–5 µM CBD impairs development of the heart, liver, kidney and eye, with the liver showing the highest sensitivity to CBD. This exposure causes liver degeneration in zebrafish embryos by modulating FABP10A, GCLC and GSR [34]. These findings underscore CBD’s potential developmental toxicity, particularly in hepatic tissues.

#### 5.2.3. Reproductive Toxicity of CBD

According to Li et al. [59], CBD exposure reduces zebrafish egg production and increases embryo malformation and mortality. In both sexes, CBD decreases gonadal indices and vitellogenin levels, while increasing hepatic indices and the proportions of premature gamete. Sex-specific estradiol/testosterone (E_2_/T) ratio inversions occur alongside testicular dysregulation of steroidogenesis genes (except *cyp11a*) and upregulation of apoptosis markers (*casp8*, *casp9*, *casp3*, and *cyt*). These findings suggest that CBD impairs reproduction through apoptosis-mediated hormonal disruptions, as evidenced by E_2_/T ratio inversions and dysregulated steroidogenesis.

Overall, while CBD shows therapeutic promise, its potential toxicity across developmental, neurological, hepatic, and reproductive systems highlights the complexity of its safety profile and the need for comprehensive studies.

### 5.3. Toxicity of CBN

Cannabinol (CBN), a degradation product of THCA via THC, was previously classified as a pharmacologically inactive cannabinoid. Recent studies have demonstrated its significant biological activities [66,67,68]. In zebrafish embryos, the LC_50_ value of CBN at 5 dpf is 1.12 mg/L (Table 3). CBN treatment (0.25–2 mg/L) exerts dose-dependent effects on morphology, behavior, physiology, and metabolome; and at concentrations exceeding 0.75 mg/L, it induces cardiac deformities, such as thinner and elongated atria, smaller ventricles, dysfunctional atrioventricular valves, and pericardial edema [35]. In another study, zebrafish embryos exposed to CBN (0.01–4 mg/L) during gastrulation (5.25–10.75 hpf) exhibits dose-dependent malformations, increased mortality, reduced locomotion, and decreased motor neuron branching. Additionally, CBN impairs the development of hair cells associated with otic vesicles and the lateral line. Furthermore, early life exposure to CBN may alter zebrafish embryonic development via cb2 receptor-mediated mechanisms [60]. These findings underscore the significant toxicological impact of CBN on zebrafish embryonic development, revealing dose-dependent cardiac and neurological impairments (Table 5).

### 5.4. Comparative Toxicity of THC, CBD, and CBN

Studies show that THC has a 10-fold higher binding affinity for cb1 and cb2 receptors than CBD [44], while CBN exhibits approximately half the affinity for cb1 and 3-fold higher affinity for cb2 receptors compared to THC. These differences in receptor binding likely contribute to the distinct pharmacological and toxicological profiles observed among these cannabinoids. Notably, CBD induces dysmorphologies at lower concentrations than THC [41], suggesting that despite its generally perceived safety in adults, it may exert greater developmental toxicity during early life stages. Consistent with this, the LC_50_ of THC is higher than that of CBD, indicating lower toxicity in zebrafish embryos. For example, Carty et al., reported that the LC_50_ for THC (3.65 mg/L) was nearly seven times higher than that for CBD (0.53 mg/L) in zebrafish exposed from the blastula to the larval stage [28].

In addition to differences in potency, CBD and THC also display distinct bioaccumulation and behavioral toxicity profiles. CBD exhibits developmental and behavioral toxicities similar to THC but at significantly lower concentrations, and it shows higher bioaccumulation, which may exacerbate its effects during prolonged exposure. [28]. Together, these findings underscore the necessity of evaluating each cannabinoid individually in toxicological studies, as receptor affinity, developmental stage, bioaccumulation, and specific phenotypic outcomes all contribute to their unique safety profiles. This nuanced understanding is particularly important when considering potential therapeutic applications or the risks associated with prenatal and early-life exposure.

## 6. Effect of SCs Using Zebrafish as an In Vivo Model

**Figure 7:**
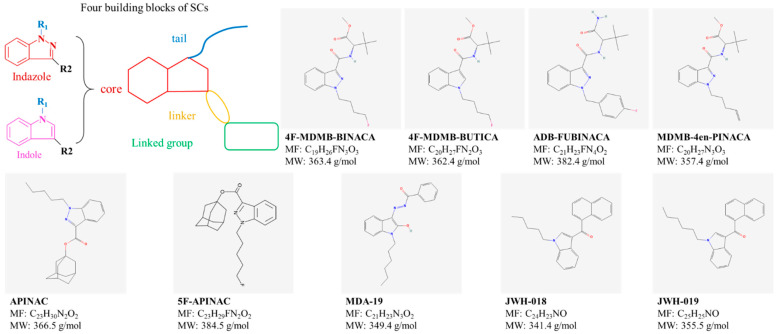
Chemical structure of synthetic cannabinoids (SCs) included in this review. Extracted from Pubchem (https://pubchem.ncbi.nlm.nih.gov/ (accessed on 25 May 2025)).

SCs are designed to mimic the psychoactive effects of THC but often exhibit significantly higher binding affinity to CB1 and/or CB2. As a result, SCs can cause more severe THC-like effects, such as increased heart rate, nausea, agitation, disorientation, and hallucinations. The European Monitoring Centre for Drugs and Drug Addiction (EMCDDA) describes the chemical architecture of SCs as consisting of four structural elements: the core, linker, linked group, and tail [69]. Figure 7 illustrates the chemical structures of SCs discussed here.

The embryotoxic effects of SCs have been insufficiently explored in the literature. This review synthesizes findings from nine studies on the developmental toxicity of SCs following embryonic exposure (Table 6), highlighting not only LC_50_ values but also diverse phenotypic outcomes. Across these studies, SCs consistently induced morphological abnormalities (e.g., spine deformities, pericardial and yolk sac edema, delayed hatching), neurodevelopmental impairments (e.g., altered locomotor activity, impaired neuronal and spinal motor neuron development, disrupted sensorimotor responses), and metabolic disturbances (e.g., changes in ROS levels and antioxidant enzyme activities). Notably, different structural classes of SCs appear to exhibit distinct toxicity patterns. For example, JWH-018, a common first-generation psychoactives SC, impairs locomotor performance in the forced light/dark test but do not affect anxiety levels or startle responses [70]. MDA-19 accelerates hatching, reduces body length, impairs swimming performance, hinders spinal motor neuron development, increases ROS, elevates SOD and CAT activity, and disrupts energy metabolism [71]. 4F-MDMB-BICA emerged on the European drug market in March 2020 and was detected in the United States in May 2020. Its LC_50_ value in zebrafish embryos is 1.932 mg/L (Table 3), Its exposure causes spine deformities, pericardial edema, impairs blood flow, yolk sac edema, and developmental delays, while modulating genes linked to apoptosis, DNA repair, and neurotransmitter systems [36]. ADB-FUBINACA, a novel indazole-based SC, was detected in major Chinese metropolitan areas at concentrations up to 1.9 ng/L [72] and exhibits a CB1 receptor binding affinity 140-fold greater than that of THC [73]. Its LC_50_ value in zebrafish embryos is 47.72 mg/L (Table 3). Using transgenic zebrafish lines *Tg (Myl7: GFP)* and *Tg (HuC: eGFP)*, Wu et al., demonstrated that ADB-FUBINACA induced abnormal cardiac morphology, impaired neuronal development, and disrupted metabolic pathways, including alanine, purine, pyrimidine metabolism, and arginine biosynthesis [37]. MDMB-4en-PINACA, first reported in Slovenia in 2018 as a yellow powder material [74], has an LC_50_ value of 37.81 µM (Table 3). It caused developmental defects, such as lordosis, pericardial edema, yolk sac edema, and delayed hatching [38]. APINAC, identified in herbal mixtures [17], impaired sensorimotor responses in zebrafish larvae [75]. Its fluorinated derivative, 5F-APINAC, with a CB1 receptor binding affinity 2~5 times greater than APINAC, altered metabolomic profiles linked to GABA, glutamic acid, dopaminergic, adrenergic, cholinergic neurotransmitter systems, and kynurenine pathway [76]. Additionally, a comparative study on the toxicity of JWH-018 and JWH-019, utilizing adult male zebrafish as an in vivo model, found that JWH-018, but not JWH-019, elicited anxiogenic effects and reduced aggression behavior [77].

Collectively, these findings demonstrate that SCs induce diverse embryotoxic effects, ranging from morphological abnormalities and neurodevelopmental impairments to metabolic disruptions, underscoring the urgent need for further research into their developmental toxicity.

## 7. Therapeutic Potential of Cannabinoids in Zebrafish Disease Models

Despite their documented adverse effects, cannabinoids exhibit a dual profile, combining both toxicological risks and therapeutic potential. Zebrafish studies have revealed that certain exposures can lead to developmental abnormalities, neurobehavioral alterations, and organ impairment, highlighting the need for careful evaluation. At the same time, these compounds act on conserved molecular targets that can be harnessed for clinical benefit. As shown in Table 7, studies using zebrafish larval disease models provide evidence of their efficacy, demonstrating diverse pharmacological actions, including antispastic, analgesic, anxiolytic, antiemetic, neuroprotective, and anti-inflammatory properties [79]. This contrast between risks and potential clinical promise underscores the importance of a balanced perspective in interpreting cannabinoid research.

### 7.1. Fin Regeneration and Anti-Apoptotic Effects

In a zebrafish embryo caudal fin amputation model, CBD crude extract (55.5% purity) accelerated fin regeneration and suppressed post-amputation apoptosis without altering mitotic activity. Pure CBD replicated these pro-regenerative effects and inhibited neutrophil infiltration at wound sites. These outcomes correlated with reduced apoptotic cell numbers and downregulated expression of interleukin-1β (IL-1β), caspase 3, and poly (ADP-ribose) polymerase (PARP) proteins. These findings suggest that CBD-mediated tissue repair involves dual mechanisms: modulation of the IL-1β/caspase 3/PARP signaling pathway and anti-inflammatory action, which collectively attenuate inflammation-driven apoptosis during wound healing [32].

### 7.2. Parkinson’s Disease

In a 6-hydroxydopamine (OHDA)-induced Parkinsonism disease zebrafish model, individual cannabinoids had no effect on OHDA-induced hypoactivity when used separately. However, three-component equimolar mixtures (e.g., CBD + CBDV + CBC, CBD + CBN + CBC, or CBD + CBDV + CBG) significantly attenuated OHDA-related motor symptoms [81]. In a haloperidol (HAL)-induced Parkinsonism model, CBD completely reverses HAL-induced motor dysfunction [82]. These findings suggest that while individual cannabinoids may lack efficacy, specific combinations or CBD alone can effectively mitigate motor impairments in Parkinsonism models.

### 7.3. Behavioral Hyperactivity and Neuroprotection

CBD alleviated behavioral hyperactivity in two distinct zebrafish models. In pentylenetetrazole (PTZ)-induced neurohyperactivity larvae, CBD exposure significantly reduced hyperactivity. In GABRA1^−/−^ mutants harboring loss-of-function mutations in the gamma-aminobutyric acid receptor subunit alpha 1 (GABRA1), CBD not only suppressed hyperactivity but also synergistically enhanced the therapeutic effects of THC, achieving greater efficacy compared to either compound administered alone [83].

### 7.4. Tuberous Sclerosis Complex (TSC)

CBD also demonstrates therapeutic potential against tuberous sclerosis complex (TSC), a rare disorder caused by autosomal-dominant sequence variations in the TSC1/TSC2 genes, characterized by tumors, epilepsy, and neurobehavioral deficits [86]. TSC1/TSC2 genes encode proteins that antagonise the mammalian isoform of the target of rapamycin complex 1 (mTORC1), a key mediator of cell growth and metabolism. In zebrafish, CBD treatment from 6 to 7 dpf reduced anxiety-like behaviors without causing sedation. Prolonged exposure starting at 3 dpf (3–10 dpf) suppressed mTOR hyperactivity, by decreasing the number and size of phosphorylated ribosomal protein S6 (rpS6)-positive cell in the brain, while maintaining normal motility and survival. These results suggest that CBD selectively modulates mTOR signaling and alleviates neurobehavioral symptoms, supporting its relevance for TSC therapy [80]. This evidence supports further exploration of CBD as a targeted treatment for TSC-related symptoms.

### 7.5. Antiseizure Activity in Epilepsy Models

Epilepsy is a major focus of cannabinoid research [87]. In the scn1Lab^−/−^ zebrafish model of Dravet Syndrome, CBD (0.6 µM), THC (1 µM), CBN (0.6 and 1 µM), and LN (4 µM) significantly suppressed seizures, with CBN exhibiting the highest efficacy. In a zebrafish epilepsy model, PTZ-induced generalized seizures, triggered by GABRA blockade, were attenuated only by CBD and THC [43]. Additionally, Pure CBD (5.7 µg/mL) and whole cannabis extracts (0.01 mg/mL) suppressed seizures more effectively than valproic acid (VPA, a commonly used antiepileptic drug), with extracts achieving comparable efficacy to CBD despite lower cannabinoid content, suggesting a potential “entourage effect” [84]. This phenomenon indicates that combining multiple cannabis-derived compounds may provide broader or more potent therapeutic benefits than CBD alone [84].

Another study revealed that CBD, CBC, and CBN effectively reduced PTZ-induced convulsions at low doses in zebrafish, with CBC showing the lowest tissue accumulation while maintaining efficacy. GPR55 partially mediated CBD’s anticonvulsant effects, and phytocannabinoid treatment altered endocannabinoid metabolism-related gene expression, suggesting modulation of endogenous cannabinoid levels as a potential mechanism underlying their antiseizure activity [57].

Collectively, these findings demonstrate the therapeutic versatility of cannabinoids in zebrafish models, particularly through anti-inflammatory, anti-apoptotic, and multi-target synergistic mechanisms, while highlighting the need to balance efficacy with safety considerations.

## 8. Perspective

Zebrafish have established themselves as a critical model system in cannabis research, harnessing their distinctive physiological and genetic attributes to elucidate the pharmacological and toxicological profiles of cannabinoids. The optical clarity of embryos/larvae permits in vivo visualization of developmental processes, enabling precise evaluation of the teratogenic effects of compounds such as THC, CBD, CBN, and synthetic cannabinoids during early life development. Their genetic tractability, enabled by advanced genome-editing tools like TALENs and CRISPR/Cas9, allows precise studies of molecular pathways influenced by cannabinoids, providing mechanistic insights into their biological effect. Adhering to the 3Rs principles, zebrafish offer an ethically and practically efficient alternative to higher vertebrate models, optimizing resource utilization in preliminary studies. Cutting-edge methodologies, including optogenetic manipulation and gene knockout strategies, enable in-depth exploration of neural circuits involved in reward processing, addiction susceptibility, and neurological disorders, which are pivotal for advancing cannabis-derived therapeutics [44].

With its scalability and physiological relevance, zebrafish supports high-throughput screening of novel cannabinoid compounds, allowing parallel evaluation of therapeutic efficacy and toxicological liability. The successful progression of several zebrafish-screened molecules into clinical trials underscores its translational value. Despite inherent differences from mammals, including metabolism, receptor homology, and routes of compound absorption, zebrafish remain a highly effective system for mechanistic studies and early-stage screening, providing critical insights that can guide subsequent research in higher vertebrates. By integrating developmental, molecular, and neuropharmacological perspectives, zebrafish provide a versatile and robust platform that bridges fundamental research and clinical applications, highlighting their indispensable role in the multidisciplinary study of cannabis. In particular, tiered testing pipelines could employ zebrafish as a first-line model, followed by validation in rodents and ultimately in clinical studies. Alongside comparative analyses of conserved pathways and biomarkers, this would further strengthen translational relevance.

## 9. Conclusions

This study provides a comprehensive analysis of the toxicological profiles of phytocannabinoids, novel synthetic cannabinoids, and the therapeutic potential of cannabinoids, utilizing zebrafish as a robust in vivo model. Our findings reveal that phytocannabinoids such as THC, CBD, CBN, and some emerging synthetic cannabinoids induce a spectrum of embryotoxic effects, encompassing developmental anomalies, neurotoxicity, hepatotoxicity, reproductive impairments, and metabolic dysregulation. These toxicities underscore the need for cautious evaluation of cannabinoid use, particularly in vulnerable populations. Nevertheless, cannabinoids and cannabinoid-derived therapeutics demonstrate significant pharmacological promise, including antispasmodic, analgesic, antiemetic, neuroprotective, and anti-inflammatory effects, with potential applications in managing complex disorders such as Parkinson’s disease, tuberous sclerosis complex, epilepsy, and tissue regeneration. These dual aspects highlight the critical balance between therapeutic benefits and toxicological risks, emphasizing the importance of further research to optimize safe and effective cannabinoid-based interventions. In particular, systematic studies are needed to address the long-term consequences of cannabinoid exposure and to clarify the toxicological profiles of emerging synthetic cannabinoids, which remain poorly understood but are of increasing public health concern.

## 10. Limitations

This review has several limitations. Firstly, the screening and data extraction were carried out by a single reviewer, which may introduce potential selection bias. Secondly, although we attempted to be comprehensive, relevant studies may still have been missed due to database coverage, search term constraints, or language restrictions. Thirdly, the included studies showed significant heterogeneity in experimental design, fish strains, developmental stages, exposure duration & concentrations, and measured endpoints, which may affect the consistency and comparability of the findings. Finally, given that zebrafish are a non-mammalian model, findings from this review should be interpreted with caution when extrapolating toxicological and therapeutic outcomes to humans. Future systematic reviews could mitigate these limitations by involving multiple independent reviewers, expanding search strategies, applying meta-analytical approaches where feasible, and integrating cross-species comparisons.

## Figures and Tables

**Table 1:** Select groups and examples of PCs, ECs, and SCs.

Cannabinoids	Category	Select Compounds	CAS NO. *	Molecular Formula (MF) *	Molecular Weight (MW, g/mol) *
Phytocannabinoids	Psychoactive	*delta*-9-tetrahydrocannabinol (Δ^9^-THC)	7663-50-5	C_21_H_30_O_2_	314.4
		Cannabinol (CBN)	521-35-7	C_21_H_26_O_2_	310.4
	Non-psychoactive	Cannabidiol (CBD)	13956-29-1	C_21_H_30_O_2_	314.4
		Cannabichromene (CBC)	20675-51-8	C_21_H_30_O_2_	314.4
		Cannabigerol (CBG)	25654-31-3	C_21_H_32_O_2_	316.5
		Cannabidivarin (CBDV)	24274-48-4	C_19_H_26_O_2_	286.4
Endocannabinoids	Major	Arachidonylethanolamine (AEA)	94421-68-8	C_22_H_37_NO_2_	347.5
		2-arachidonoyl glycerol (2-AG)	53847-30-6	C_23_H_38_O_4_	378.5
	Minor	Noladin ether	222723-55-9	C_23_H_40_O_3_	364.6
		Virodhamine	287937-12-6	C_22_H_37_O_2_	347.5
		N-Arachidonyl dopamine (NADA)	199875-69-9	C_28_H_41_NO_3_	439.6
		Oleamide	301-02-0	C_18_H_35_NO	281.5
Synthetic cannabinoids	/	MDA-19	1048973-47-2	C_21_H_23_N_3_O_2_	349.4
		JWH-018	209414-07-3	C_24_H_23_NO	341.4
		JWH-019	209414-08-4	C_25_H_25_NO	355.5
		APINAC	2219331-93-6	C_23_H_30_N_2_O_2_	366.5
		5F-APINAC	2365471-88-9	C_23_H_29_FN_2_O_2_	384.5
		ADB-FUBINACA	1445583-51-6	C_21_H_23_FN_4_O_2_	382.4
		MDMB-4en-PINACA	2504100-70-1	C_20_H_27_N_3_O_3_	357.4
		4F-MDMB-BINACA	2390036-46-9	C_19_H_26_FN_3_O_3_	363.4
		4F-MDMB-BUTICA	2682867-53-2	C_20_H_27_FN_2_O_3_	362.4

* Extracted from Pubchem (https://pubchem.ncbi.nlm.nih.gov/ (accessed on 15 May 2025)).

**Table 2:** Cannabinoid-containing medicinal products approved by the FDA and/or EMA.

Drug	Active Pharmaceutical Ingredient	Medical Conditions	Company	Country and Year Approved
Epidiolex	Nabiximols (CBD)	Seizures associated with Lennox–Gastaut syndrome and Dravet syndrome	Greenwich Biosciences	USA 2018, EU 2019
Sativex	2.7 mg/mL of Δ^9^-THC and 2.5 mg/mL of CBD (approx. equal quantities of THC and CBD)	Neuropathic pain, spasticity, overactive bladder, and other symptoms of multiple sclerosis	GW Pharmaceuticals	Canada 2005, UK 2010, Spain 2010, Germany 2011, Denmark 2011, Sweden 2012, Australia 2012
Marinol	Dronabinol (synthetic Δ^9^-THC)	HIV/AIDS-induced anorexia and chemotherapy-induced nausea and vomiting	Unimed Pharmaceuticals	USA 1985
Cesamet	Nabilone (synthetic Δ^9^-THC)	Nausea, Mutiple sclerosis, Fibromyalgla	Valeant Pharmaceuticals	USA 1985/2006
Syndros	Dronabinol (synthetic Δ^9^-THC in liquid formulation)	Nausea and vomiting caused by chemotherapy, loss of appetite	Insys Therapeutics	USA 2006

Full name: FDA: Food and Drug Administration; EMA: European Medicines.

**Table 3:** Median lethal concentrations (LC_50_) of PCs and SCs in zebrafish embryos/larvae.

Cannabinoid	Concentrations for LC_50_ Calculation	LC_50_	Refs.
THC	0.3, 0.6, 1.25, 2.5, 5 mg/L	3.65 mg/L (11.61 µM)	[28]
	0.3–9.6 mg/L	3.37 mg/L (10.72 µM)	[29]
	0.3–9.6 mg/L	3.4 mg/L (10.81 µM)	[30]
	1, 1.25, 1.5, 2 mg/L	1.54 mg/L (4.9 µM)	[31]
CBD extract	0.625, 1.25, 2.5, 5, 12.5, 25 mg/L	48 hpf: 4.4 mg/L	[32]
		72 hpf: 3.7 mg/L	
CBD	0.07, 0.1, 0.3, 0.6, 1.25 mg/L	793.28 µg/L (2.52 µM)	[33]
	0.07, 0.1, 0.3, 0.6, 1.25 mg/L	0.53 mg/L (1.69 µM)	[28]
	0.1, 0.5, 1, 5, 10, 25, 50 µM	24 hpf: 49.33 µM (15.5 mg/L)	[34]
		48 hpf: 32.25 µM (10.14 mg/L)	
		72 hpf:16.98 µM (5.33 mg/L)	
		96 hpf: 5.883 µM (1.85 mg/L)	
CBN	0.25–10 mg/L	1.12 mg/L (3.61 µM)	[35]
4F-MDMB-BUTICA	0.15, 0.3, 0.6, 1.2, 2.4, 4.8 mg/L	120 hpf: 1.932 mg/L (5.33 µM)	[36]
ADB-FUBINACA	10, 20, 40, 50, 60, 80, 100 mg/L	96 hpf: 47.72 mg/L (124.79 µM)	[37]
MDMB-4en-PINACA	0.001–10 µM	37.81 µM (13.51 mg/L)	[38]

**Table 4:** Effects of Δ^9^-THC on the whole organism in zebrafish (*Danio rerio*) in-vivo studies.

Zebrafish Strain	Concentrations	Initial Exposure Stage	Exposure Duration	Phenotypes	Refs.
Not provided	1, 2, 5, 10 mg/L	Blastula (4.5 hpf)	4.5–24 hpf	Reduce spontaneous tail muscle twitch, curved trunks and bulbous-tipped tails	[27]
Not provided	0.3, 0.6, 1.2, 2.4 mg/L	Larval (108 hpf)	1, 4, 12 h	Dose-dependent dual-phase locomotor response, with activation at low doses and inhibition at high doses	[29]
		24 hpf	96 h	Trigger habituation in basal/recovery phases across all concentrations, with only 1.2 mg/L stimulating activity	
AB/TU hybrids	0.1, 0.5, 1.5, 2 µM	Larval (5 dpf)	/	Distinct behavioral patterns and concentration response profiles	[39]
*Tg (fli1:eGFP)*	0.3125, 0.625, 1.25, 2.5, 5 mg/L	Blastula (2 hpf)	2–96 hpf	LC_50_: 3.65 mg/L; edemas, curved axis, eye/snout/jaw/trunk/fin deformities, swim bladder distention, behavioral abnormalities	[28]
	0.08, 0.4, 2 µM	Gastrula (6 hpf)	6–14/24/48/72/96 hpf	Cause biphasic effects on longevity, inflammation, and reproduction in aged fish	[40]
*Tg (fli1: eGFP)*	0.024, 0.12, 0.6 mg/L	Gastrula (6 hpf)	6–96 hpf	Reduce fecundity in adults. Did not cause notable morphological abnormalities in either F0 or F1 generations	[41]
TL	6 mg/L	Gastrula (5.25 hpf)	5.25–10.75 hpf	Reduce axonal diameter of Mauthner cells (M-cell), alters escape response properties	[42]
TL strain, scn1Lab^−/−^, scn1Lab^+/−^	1, 4 µM	Larval (5 dpf)	120–144 hpf	Reduce seizure behavior in chemically induced and scn1a-mutant zebrafish	[43]
CaMPARI transgenic/ Casper	2, 3, 4, 6 mg/L	Zygote (0.5 hpf)	0.5–10 hpf	Reduce neural activity and locomotion	[44]
TL	10 mg/L	Larval (5 dpf)	0.5 h	Alter motor neuron-muscle communication and motor behaviors	[45]
TL	2, 4, 6, 8, 10 mg/L	Gastrula (5.25 hpf)	5.25–10.75 hpf	Reduce heart rates, axial malformations and shorter trunks, alter synaptic activity at neuromuscular junctions, change in branching patterns and a reduction in the number of axonal branches in the trunk musculature	[46]
TL	0.001, 0.01, 0.1, 0.5, 1, 10, 20 mg/L	Gastrula (5.25 hpf)	5.25–10.75 hpf	Reduce spontaneous coiling of 1-dpf embryos, reduce swimming after touch-evoked responses and basal swimming in 5-dpf larvae. Reduce coiling activity of F1 embryos, reduce swimming after touch-evoked responses of 1-dpf F1 embryos.	[47]
Not provided	100 µM	Adult	1 h	Impairs spatial but not associative memory function, activation of extracellular signal-regulated kinases signaling in the lateral pallium	[48]
Not provided	100 nM	Adult	1 h	Inhibit acquisition of fear learning	[49]
Short-fin	30, 50 mg/L	Adult	20 min	Produce an anxiogenic-like reduction of top swimming, paralleled with a slower, continuous bottom swimming	[50]
EK-WT	40 nM; 1, 2 µM	Adult	20 min	Induce psychosis-like behavioral stereotypy	[51]

**Table 5:** Effects of CBD and CBN on the whole organism in zebrafish (*Danio rerio*) in-vivo studies.

Zebrafish Strain	Concentrations	Initial Exposure Stage	Exposure Duration	Phenotypes	Refs.
CBD					
Not provided	5, 20, 70, 150, 300 µg/L	Zygote	96 h	No malformation, do not alter biochemical activity; increases the motor activity at 24 hpf, but not at 48 hpf.	[52]
	0.5, 1, 5, 10 mg/L	Larval (4~5 dpf)	30 min	0.5 and 10 mg/L reduce movement velocity and the total distance	[53]
AB/TU	up to 3.14 mg/L	Larval (2 dpf)	2–5 dpf	>2.5 µM led to higher levels of toxicity to the larvae	[54]
*Tg (fli1:eGFP)*	0.075, 0.15, 0.3, 0.6, 1.2 mg/L	Blastula (2 hpf)	2–96 hpf	LC_50_: 0.53 mg/L; Edemas, curved axis, eye/snout/jaw/trunk/fin deformities, swim bladder distention, behavioral abnormalities	[28]
TU	0.1 0.5, 5.0, 10 mg/kg (i.p.)	Larval (3 dpf)	1 h before analysis	Inverted U-shaped dose–response curve with 0.5 mg/kg reducing the anxiety. 5 mg/kg causes memory impairment.	[55]
CaMPARI transgenic/Casper	1.5, 2, 3 mg/L	0.5 hpf	0.5–10 hpf	Dramatically reduce neural activity and locomotor activity.	[44]
*Tg (fli1: eGFP)*	0.006, 0.03, 0.15 mg/L	Gastrula (6 hpf)	6–96 hpf	Did not cause notable morphological abnormalities in either F0 or F1 generations	[41]
AB	0.25, 0.5, 0.75, 1, 1.25, 1.5 mg/L for acute toxicity; 0.1, 0.2 mg/L for reproductive system development	Gastrula (4 hpf)	4–7 dpf	LC_50_: 793.28 µg/L; developmental toxicity, lethal toxicity, and reproductive inhibition	[33]
TL	1, 2, 3, 4 mg/L	Gastrula (5.25 hpf)	5.25–10.75 hpf	Reduce heartbeat rates, axial malformations, shortened trunk length, and impair synaptic activity at neuromuscular junctions, along with altered axonal branching patterns and decrease axonal branches number in trunk musculature.	[46]
TL	3 mg/L	Gastrula (5.25 hpf)	5.25–10.75 hpf	Reduce hatching and survival rates and suppress Shh pathway activity, impair swimming activity.	[56]
Not provided	0.01, 0.05, 0.1, 0.5, 1, 5, 10, 25, 50 µM	Gastrula (6 hpf)	6–96 hpf	Reduce heartbeat rates, induce pericardial edema, and reduce eye area,	[34]
AB	0.1, 0.25, 0.5, 1.25 mg/L	Blastula (2 hpf)	6, 48 h	Promote fin regeneration and inhibit neutrophil accumulation in a dose-dependent manner.	[32]
AB	1, 2, 4 µM	Larval (6 dpf)	30 min	Significantly inhibit PTZ-induced hyperactivity at 2 and 4 µM concentrations.	[57]
Not provided	40 mg/L	Adult	30 min	Decrease swimming speed and swimming distance, affect immune gene expression	[58]
AB	500, 600, 700, 800, 900 µg/L	Adult	96 h	Both sexes show lower gonadosomatic indices (GSI), higher hepatosomatic indices (HSI), immature gametes, and reduce vitellogenin (VTG) levels. E2/T ratio decreases in female while increases in males. Apoptosis-related genes are upregulated in the brain, gonad, and liver.	[59]
CBN					
TL	0.01, 0.1, 0.5, 1, 2, 3, 4 mg/L	Gastrula (5.25 hpf)	5.25–10.75 hpf	Causes dose-dependent malformations, higher mortality, reduced locomotion, and impaired motor neuron branching. It also disrupts hair cell development in the otic vesicles and lateral line, weakening sound response.	[60]
AB	0.25, 0.75, 1.0, 1.125, 1.2, 1.25, 2.0 mg/L	Somite (26 hpf)	24–120 hpf	Pericardial edema, yolk sac anomalies and tail bending, increases total movement distance and velocity	[35]

**Table 6:** Effects of synthetic cannabinoids (SCs) on the whole organism in zebrafish (*Danio rerio*) in-vivo studies.

Type	Strain	Concentration	Initial Exposure Stage	Exposure Duration	Phenotypes	Refs.
4F-MDMB-BINACA	AB	25 µM	Larval (4 dpf)	24 h	Impaired liver development	[78]
4F-MDMB-BUTICA	AB	0.15, 0.3, 0.6, 1.2, 2.4, 4.8 mg/L	Blastula (3 hpf)	3–24 hpf (acute); 3–120 hpf (subacute)	Caused embryonic deformities, including spine formation, pericardial edema, impaired blood flow, yolk sac edema, delayed development. Induced hypoactivity in response to stimulus. altered the transcriptional expression levels of apoptosis, DNA repair, dopamine, serotonin, γ-aminobutyric, and behavior-related genes	[36]
ADB-FUBINACA	AB; *Tg (Myl7:GFP)*, *Tg (HuC:eGFP)*	10, 20, 30 mg/L	Blastula (4 hpf)	4–96 hpf	Reduced heartbeat, shorter body length, spinal deformation, and pericardial edema, cardiac developmental defects, impaired motor activity, disrupted neuronal development, elevated ROS and MDA, dysregulated immune-related genes, disruptions in pathways related to alanine, purine, pyrimidine metabolism, arginine biosynthesis.	[37]
MDA-19	AB; *Tg (hb9: GFP)*	1, 10, 20 mg/L	/	5 days	Accelerated hatching, reduced body length without affecting mortality or malformation, resulted in diminished swimming ability and reduced activity time, impaired development of spinal motor neurons, increased ROS, elevated SOD and CAT, affected energy metabolism	[71]
JWH-018	Not provided	3 µM	Pharyngula (28 hpf)	1–6 dpf	Impaired locomotion during the forced light/dark test	[70]
	AB	0.01, 0.05, and 0.25 µg/g	Adult (intraperitoneally, i.p.)	/	Dose-dependent anxiogenic effects and lower aggression behavior, activated the CB1R-dependent extracellular signal-regulated kinase 1 and 2. Did not affect the vertical movement distance.	[77]
JWH-019	AB	0.01, 0.05, and 0.25 µg/g		/	Did not change the movement trace line and vertical movement distance.	
APINAC	Not provided	0.001, 0.1, 1, 10 µM	Larval (6 dpf)	/	Reduced visual motor response, impairment of spontaneous motor and sensorimotor behavior	[75]
5F-APINAC	Not provided	0.001, 0.01, 0.1, 1.0 10 µM	Larval (6 dpf)	4 h, 96 h	Morphological and developmental alterations, induced metabolomic alterations	[76]
MDMB-4en-PINACA	Not provided	0.001, 0.01, 0.1, 1, 10 µM	Blastula (1.5 hpf)	1.5–4 dpf	Positive correlation between exposure concentration and lethal effects, including lack of heartbeat, lack of somite formation, pericardial edema, and yolk edema.	[38]

Full name: 4F-MDMB-BINACA: methyl 2-[1-(4-fluorobutyl)-1H-indazole-3-carboxamido]-3,3-dimethylbutanoate; 4F-MDMB-BUTICA: methyl 2-(1-(4-fluorobutyl)-1H-indole-3-carboxamido)-3,3-dimethylbutanoate; ADB-FUBINACA: N-(1-amino-3,3-dimethyl-1-oxobutan-2-yl)-1-(4-fluorobenzyl)-1H-indazole-3-carboxamide; MDA-19: BZO-HEXOXIZID (N’-[(3Z)-1-(1-hexyl)-2-oxo-1,2-dihydro-3H-indol-3-ylidene; JWH-018: 1-pentyl-3-(1-naphthoyl)-indole; JWH-019: 1-Hexyl-3-(naphthalen-1-oyl) indole; APINAC: 1-adamantyl 1-pentylindazole-3-carboxylate; 5F-APINAC: adamantan-1-yl 1-(5-fluoropentyl)-1H-indazole-3-carboxylate; MDMB-4en-PINACA: methyl (S)-3,3-dimethyl-2-(1-(pent-4-en-1-yl)-1H-indazole-3-carboxamido)butanoate).

**Table 7:** Therapeutic potential of cannabinoids in zebrafish-induced disease models.

Cannabinoids	Concentrations	Disease Models	Effects	Refs.
CBD crude extract	0.1, 0.25, 0.5, 1.25 mg/L	Caudal fin amputation model (amputated at 3 dpf, observations at 48- and 72-h post-amputation)	Accelerated fin regeneration and suppressed post-amputation apoptosis	[32]
CBD	1.25 µM	Tuberous sclerosis complex	Reduced anxiety-like behaviors without sedation	[80]
CBD, CBC, CBDV, CBG, CBN	CBD, CBN, and CBDV: 0.25–4 µM CBC: 0.1–3 µM CBG: 0.25–3 µM	6-hydroxydopamine (OHDA)-induced Parkinson’s disease	Individual cannabinoids had no effect on OHDA-induced hypoactivity. However, three-component equimolar mixtures (e.g., CBD + CBDV + CBC, CBD + CBN + CBC, or CBD + CBDV + CBG) significantly attenuated OHDA-related motor symptoms	[81]
CBD	1, 5, 10 mg/L	Haloperidol induced Parkinsonism model	CBD and ropinirole reversal haloperidol-induced motor dysfunction, CBD was more effective than ropinirole.	[82]
CBD, THC	CBD: 1, 1.5, 2 µM THC: 1.5, 2, 3 µM	PTZ-induced neurohyperactivity model; GABRA1^−/−^ mutants model	CBD alleviated behavioral hyperactivity in two different zebrafish models. It not only calmed hyperactivity but also worked synergistically with THC to amplify therapeutic outcomes, surpassing the effectiveness of either compound used independently.	[83]
CBD, THC, CBVD, CBN, linalool (LN)	CBD: 0.3, 0.6, 1.0 µM THC: 1.0, 4.0 µM CBVD: 0.3, 0.6, 1.0 µM CBN: 0.3, 0.6, 1.0 µM LN: 0.3, 0.6, 1.0, 4.0 µM	(scn1Lab^−/−^) Dravet Syndrome model; PTZ-induced epilepsy model	CBD (0.6 µM), THC (1 µM), CBN (0.6, 1 µM), and LN (4 µM) significantly suppressed seizures, with CBN exhibiting maximal efficacy. Only CBD and THC attenuated PTZ-induced hyperactivity	[43]
CBD; Whole cannabis extracts	6, 9, 12, 15, 18 µM	PTZ-induced epilepsy model	Pure CBD (5.7 µg/mL) and whole cannabis extracts (0.01 mg/mL) outperformed valproic acid (VPA, a commonly used antiepileptic drug) in seizure suppression, with extracts achieving comparable efficacy to CBD despite lower cannabinoid content	[84]
CBD, CBC, CBN	CBC: 1, 2, 4 µM CBN: 1, 2, 4 µM	PTZ-induced epilepsy model	CBD, CBC, and CBN effectively reduced PTZ-induced convulsions at low doses in zebrafish, with CBC showing the lowest tissue accumulation while maintaining efficacy. CBN inhibited PTZ-induced hyperactivity, with 2 and 4 µM doses being statistically significant. CBC inhibited PTZ-induced hyperactivity in a dose-dependent manner, with 1, 2, and 4 µM doses being statistically significant	[57]
THCV, CBD	THCV: 5 µM CBD: 5 µM	Naive larvae	CBD and THCV reduced AdipoRed staining of larvae yolk sacs within 24 h and 48 h respectively	[85]

## Data Availability

Data presented in this paper were sourced from the online publications.
